# Herbivorous sea urchins (*Echinometra mathaei*) support resilience on overfished and sedimented tropical reefs

**DOI:** 10.1038/s41598-024-52222-0

**Published:** 2024-02-15

**Authors:** Caitlin R. Fong, Nefertiti Smith, Elijah Catalan, Blanca Alvarez Caraveo, Paul H. Barber, Peggy Fong

**Affiliations:** 1 NCEAS, Santa Barbara, USA; 2 UCI, San Diego, USA; 3 Hampton University, Hampton, USA; 4 Howard University, Washington DC, USA; 5 UCLA, Los Angeles, USA; 6 Barnard College, New York, USA

**Keywords:** Ecology, Ecology, Environmental sciences, Ocean sciences

## Abstract

Human impacts are dramatically changing ecological communities, motivating research on resilience. Tropical reefs are increasingly undergoing transitions to short algal turf, a successional community that mediates either recovery to coral by allowing recruitment or transitions to longer turf/macroalgae. Intense herbivory limits turf height; subsequently, overfishing erodes resilience of the desirable coral-dominated reef state. Increased sedimentation also erodes resilience through smothering and herbivory suppression. In spite of this critical role, most herbivory studies on tropical reefs focus on fishes, and the contribution of urchins remains under-studied. To test how different herbivory and sedimentation scenarios impact turf resilience, we experimentally simulated, in situ, four future overfishing scenarios derived from patterns of fish and urchin loss in other reef systems and two future sedimentation regimes. We found urchins were critical to short turf resilience, maintaining this state even with reduced fish herbivory and increased sediment. Further, urchins cleared sediment, facilitating fish herbivory. This study articulates the likelihood of increased reliance on urchins on impacted reefs in the Anthropocene.

## Introduction

Ecological communities are changing at an unprecedented rate, driven by increasing and intensifying human impacts^1^, motivating research on the underpinnings of community resilience. Globally, community changes affect ecological structure and function as well as ecosystem goods and services^2^. The outcome of community shifts include changes in species composition^3^, trophic support^4,5^, and biogeochemical cycling^6,7^. However, resilient communities, by definition, maintain community structure and function, either by resisting change or recovering rapidly^8,9^. While both components of resilience are critical to maintaining ecosystem function, understanding a community’s capacity to resist change, and the species or processes that drive that resistance, is critical as communities face increasing and accelerating stressors.

Human impacts are causing coral reefs to decline globally^10,11^. When coral is lost, either naturally or due to anthropogenic pressures, short turf is an early successional community that can mediate either recovery to coral or transitions to algal dominance^5,10,12–14,14,15^. Recent evidence suggests that the height of turf can determine the community trajectory; short turf allows recovery to coral whereas tall turf can trap sediment and lead to a shift to macroalgae^15–17^ or an algal-dominated alternative stable state (*sensu*^18^). Thus, understanding the factors that regulate short turf resilience is essential to managing reefs.

Short turf is highly productive, an abundant part of healthy reefs, and the preferred resource for many herbivores^19–21^. Short turf is maintained by strong herbivory and low sedimentation^5,15^; if these forces weaken, turf can lose resilience and shift to taller turf or macroalgae^4,21^. Notably, some research suggests transitions to tall turf may be an early indicator of community degradation^16,18^. Thus, understanding the response of short turf to future scenarios that alter herbivory and sedimentation is critical to informing management plans to protect resilience.

The preponderance of research on herbivory on coral reefs focuses on fishes^5,10,14,15,22–27^. Ample experimental evidence demonstrates the capacity of herbivorous fishes to control algal accumulation and prevent transitions to macroalgae^5,14,15,22,25^. In contrast to temperate systems where the importance of urchin grazing is well established^28^, evidence for the importance of herbivorous urchins on tropical reefs is sparse, with most research from the Caribbean and Indian Oceans, and little empirical work in the Pacific^12,29,30^. Although less studied than herbivorous fishes, evidence supporting the role of urchins in regulating turf communities on tropical reefs is compelling. For example, Caribbean reefs rapidly transitioned to macroalgae following the urchin die off in the 1980s^12^, indicating declines in both fishes and urchins can have a profound impact on turf resilience on reefs.

Increased sedimentation also erodes the resilience of short algal turf on tropical reefs, while enhancing resilience of collapsed reef states such as long sediment trapping turfs, and sedimentation is likely to continue increasing in the Anthropocene^15,21,31–33^. Typically, on short turf communities (< 1 mm tall), sediment loads are nearly undetectable, and at most 0.5 mm deep^34^; however, even small changes in sediment load can restructure turf communities and decrease provisioning to herbivorous fishes^5,15,35,36^. Sediment deposited on turf largely comes through run-off and loading from developed watersheds as well as resuspension and resettlement. Notably, terrigenous sediments arise from coastal development while resuspension of coral-derived sediments via resuspension and resettlement is general to coral reefs, especially lagoonal environments^36^. For example, storms can pulse terrestrial sediment into tropical reefs^33^ as well as drive resuspension of benthic sediment, which is likely a mix of marine (calcium carbonate) and terrestrial sources^36,37^. Sediment can both smother corals^31,32^ and reduce coral recruitment^16^, preventing coral recovery. Sediment also decreases turf productivity^21^ and fish herbivory rates^35,38–42^. Importantly, there is a feedback loop where longer turf algae traps more sediment, which then grows even longer^21^. In many tropical regions, storminess is likely to increase with climate change^43^. Thus, understanding the importance of increased sedimentation on reefs may provide insights into short turf resilience both currently and in the future.

We employ scenario analysis to explore one aspect of turf resilience^8^, its resistance to a trajectory toward a taller, sedimented state. Specifically, we test the effects of herbivory and sedimentation on turf community resistance with a fully-crossed manipulative field experiment on naturally-occurring short turf communities on a fringing reef in Moorea, French Polynesia between July 02, 2018 and July 17, 2018. Specifically, we modelled four different future scenarios of overfishing of herbivorous fishes and urchins (1) status quo, (2) moderate overfishing and no urchins, (3) severe overfishing and intact urchin community, and (4) severe overfishing and no urchins – see methods at end for a complete description) each with or without increased sediment loads.

## Results

**Figure 1:**
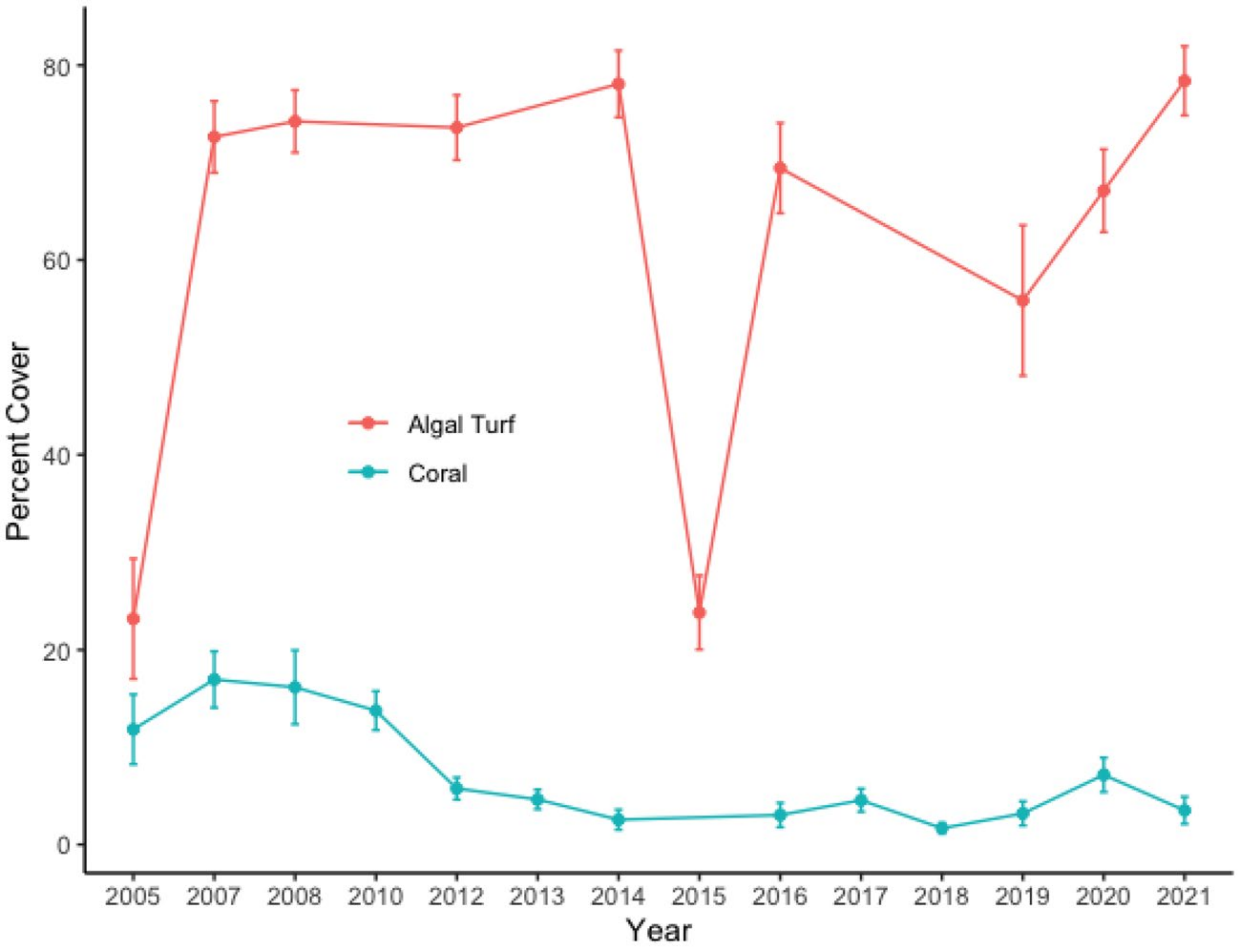
Percent benthic cover of turf and coral on the experimental reef off of the town of Maharepa, Moorea, French Polynesia. Errors are ± SE, N = 25. Moorea Coral Reef Long Term Ecological Research data.

To set the context for our experimental manipulations, we used benthic cover data at this site from the Moorea Coral Reef Long Term Ecological Research project*.* In 2018 turf algae dominated our study site (55.8 ± 7.7% SE) while coral was rare (3.2 ± 1.2%) (Fig. 1). This was a pattern dating back to 2005.

**Figure 2:**
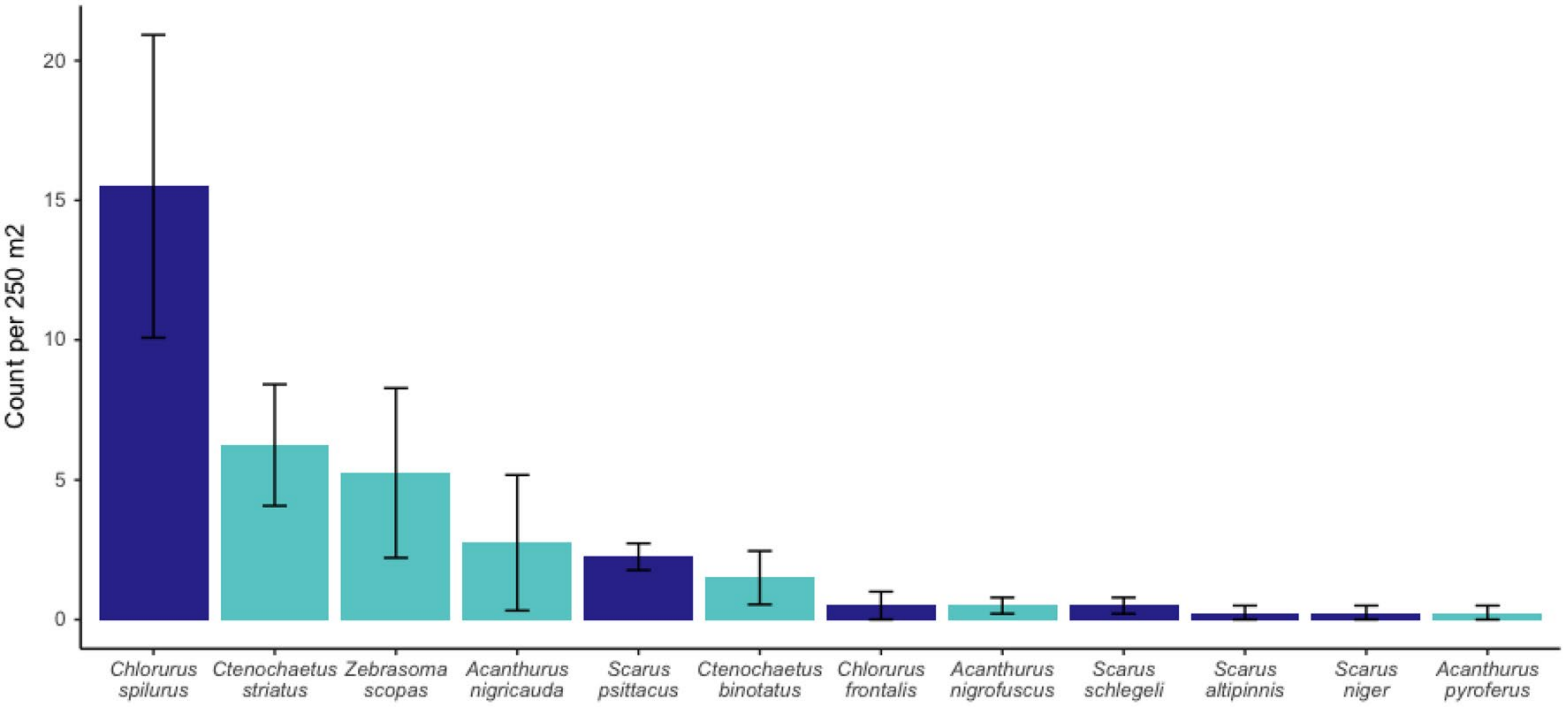
Abundance of herbivorous fishes on the experimental reef off of the town of Maharepa, Moorea, French Polynesia. Dark blue bars are Labridae (subfamily Scarinae), light blue bars are Acanthuridae. Bars are means ± SE, N = 5. Annual means for 2019 from Moorea Coral Reef Long Term Ecological Research data.

We also used MCR LTER data to contextualize the herbivore populations in our experimental site. In 2019, our site had two species of urchins, *Echinometra mathaei* (> 96% of the urchin community) and *Echinothrix calamaris* (< 55% of the urchin community); mean density of urchins was 1.5 ± SE 0.4 urchins^44^ per m^2^, a ‘low density’ state for this system^45^. In 2019, herbivorous fish biomass was 2.25 ± 1.5 kg (mean ± se) per 100 m^2^ and abundance was 14.3 ± 2.1 (mean ± se) herbivorous fishes^46^ per 100 m^2^. The four dominant herbivorous/detritivorous fishes—*Chlororus spilurus, Ctenochaetus striatus, Zebrasoma scopas,* and *Acanthurus nigricauda—*comprised ~ 60% of the community (Fig. 2). Acanthuridae and Labridae (subfamily Scarinae) were similar in abundance (45 and 55% of the community, respectively).

**Figure 3:**
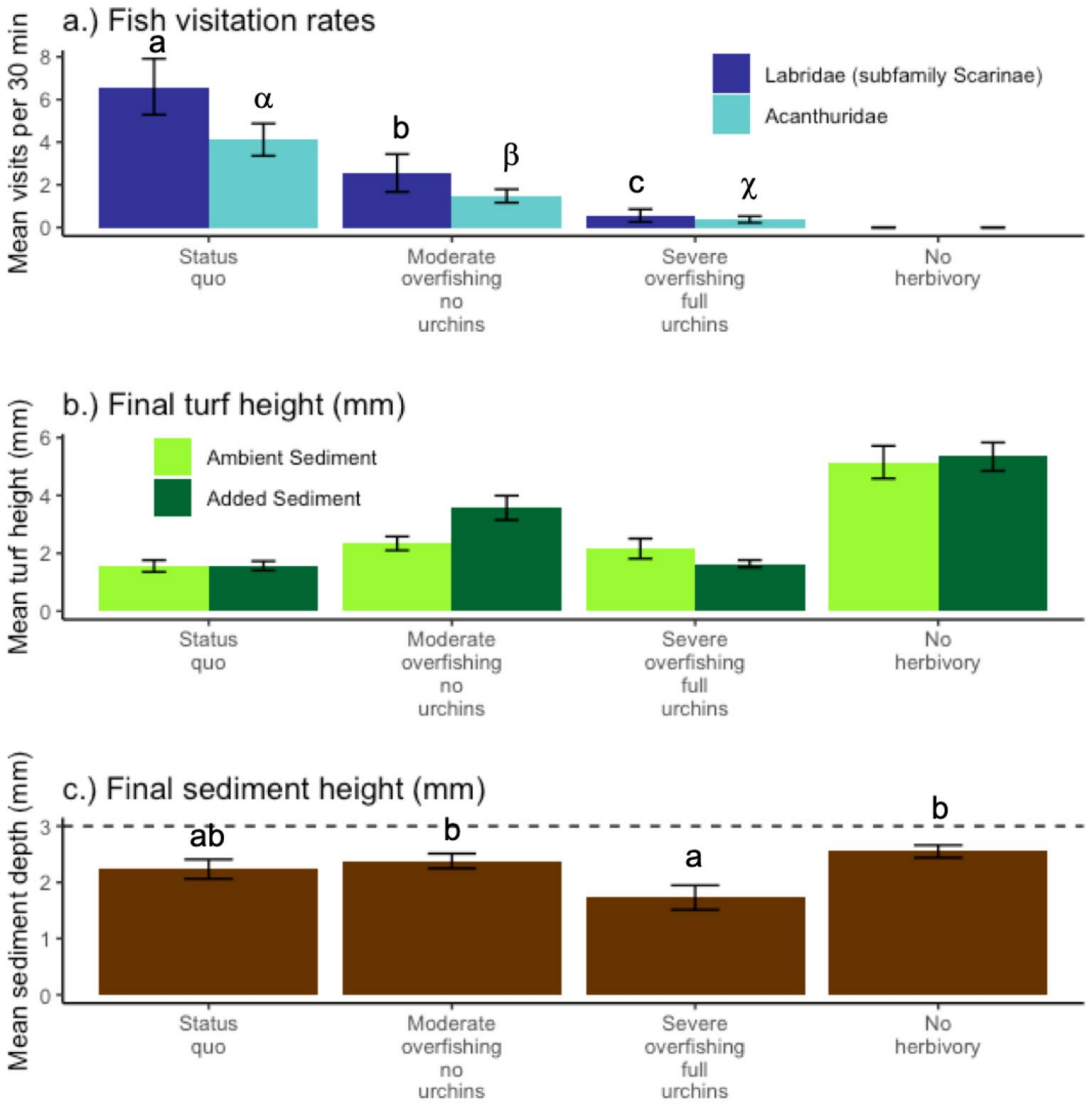
(**a**) Visitation rates of herbivorous fishes across all 4 scenarios in replicate 30-min focal periods. (**b**) Final turf height (mm) after 15 days of treatment. (**c**) Final sediment depth (mm) after 15 days of treatment and three days after the last reapplication. Dotted horizontal line is the depth of sediment we added for the sediment addition scenario . Bars are mean ± SE.

Visitation of experimental plots by both Labridae (subfamily Scarinae) and Acanthuridae was significantly and progressively reduced across cage types, simulating different scenarios of herbivorous fish loss (Fig. 3a, Table 1). Reductions were similar for both families, with the moderate overfishing no urchin scenario reducing fish visitation rates relative to the control (e.g. status quo scenario) by approximately 60% and 64%, respectively (Fig. 3a). The severe overfishing full urchin scenario had further reduction in visitation rates, at approximately 10% of the status quo scenario. No herbivorous fishes were observed in the no herbivory scenario. Because herbivorous urchins are nocturnal, we did not quantify visitation rates; however, we qualitatively confirmed that urchins could access the status quo and severe overfishing full urchin scenarios on a night dive, and did not observe any urchins in the two no urchin scenarios.

**Table 1:** Summary statistics for Kruskal–Wallis test comparing mean fish visitation rates across the 4 herbivory scenarios. Follow up pairwise Wilcox tests with corrections indicate every scenario was significantly different for both families.

	df	Chi-Square	*p* value
Acanthuridae	3	35.042	< 0.0001
Labridea (tribe Scarinae)	3	34.006.13	< 0.0001

Final turf height was strongly driven by herbivory scenario, and we found a marginally significant interaction between herbivory and sediment scenarios (Fig. 3b, Table 2). Overall, the status quo scenario had the shortest turf, with no difference in means between sediment scenarios. The severe overfishing full urchin scenario had the second shortest turf, and was 38% and 5% taller than the status quo scenario for ambient and added sediment scenarios, respectively. The moderate overfishing no urchin scenario had turf 50% and 120% taller than the status quo scenario for ambient and added sediment scenarios, respectively. Thus, in this scenario, turf height with added sediment was taller than with ambient sediment. which was the opposite pattern from that in the severe overfishing and full urchin scenario. Thus, sediment deterred fish herbivory when urchins were excluded, yet when urchins had access, there was no effect of sediment on turf height. Finally, the no herbivory scenario had the tallest turf, and, like the status quo scenario, no difference in turf height between added and ambient sediment scenarios. The no herbivory scenario had turf more than three times taller than the status quo scenario. Overall, the difference in pattern across sediment scenarios generated the marginally significant interaction.

**Table 2:** Summary statistics for final turf height. Data met assumptions of normality and homogeneity of variance after a log transformation (Shapiro test: W(80) = 0.99036, *p* value = 0.8169; Bartlett test: alpha = 0.05, df = 7, 73, *p* value = 0.6241).

	df	SS	MSE	F-value	p-value
Sediment	1	0.1183	0.1183	0.8780	0.35189
Herbivory scenario	3	17.6526	5.8842	43.6876	< 0.0001
Herbivory scenario × Sediment	3	0.9238	0.3079	2.2862	0.08594
Residuals	72	9.6975	0.1347		

Across all scenarios, sediment depth in the sediment addition scenarios decreased during the 3 days after the last 3 mm deep application; however, retention varied across scenarios (Fig. 3c, Table 3). The severe overfishing full urchin scenario retained significantly less sediment than either of the scenarios where urchin access was restricted. In contrast, retention did not differ between the two scenarios where urchins were present (Tukey HSD), although the status quo also did not differ from either urchin removal scenario. The severe overfishing full urchin scenario retained almost 60% of the applied sediment, with a final depth of 1.73 ± 0.22 mm (mean ± se). This is approximately 2/3 the depth of the no herbivory scenario, which retained approximately 85% of the application. Similarly, the moderate overfishing no urchin scenario retained approximately 80% of the sediment we applied, and had 40% deeper sediment than the severe overfishing full urchin scenario. The status quo scenario was statistically indistinguishable from the other three scenarios. This scenario retained approximately 75% of the sediment we applied, for a final sediment depth of 2.23 ± 0.17 (mean ± se).

**Table 3:** Summary statistics for final sediment depth. Data met assumptions of normality and homogeneity of variance (Shapiro test: W(40) = 0.94892, *p* value = 0.06963; Bartlett test: alpha = 0.05, df = 3,37, *p* value = 0.2169).

	df	SS	MSE	*F* value	*p* value
Herbivory scenario	3	3.7403	1.24676	4.6812	0.007329
Residuals	36	9.5881	0.26634		

## Discussion

We found that urchins have the potential to play a critical role in the resilience of turf communities on tropical reefs with increased sedimentation and reduced fish abundance, two likely future scenarios for coral reefs globally^21,31–33^. Urchins kept turf closely-cropped, even in the context of severely reduced fish herbivory and heightened sediment load. Importantly, urchins also significantly reduced sediment loads on turf. Because sediment reduces fish herbivory^35,39,39–42^, sediment removal during urchin herbivory likely has an added positive effect of facilitating fish herbivory. Combined, these results demonstrate the potential for increased reliance on herbivorous urchins for the resilience of tropical reefs in the Anthropocene. We posit that too few urchins may not provide any ecological redundancy to fish herbivores, and may be ineffective at clearing sediment to promote fish herbivory. But a cautionary tale is the likely cost of too many urchins is increased bioerosion and reduced coral recruitment. Thus, empirical research should explore theses future scenarios to determine what intermediate value of urchins is optimal for conservation and restoration efforts.

Urchins kept turf closely cropped, a process that may be especially critical in the context of overfishing. Most evidence for the importance of urchin grazers comes from studies of Caribbean and African reefs when herbivorous fishes were decimated by overfishing (Caribbean^12,29,47,48^, African^30,49^). Our work adds to this evidence that overfishing reveals an emergent functional role for urchins. For example, our scenario of severe overfishing and no urchins modeled what happened in the Caribbean where, after decades of overfishing, resilience maintained by urchins was eroded following the massive urchin die off in the 1980s^10,12,47,48,50,51^. Further, in Kenya, recent experimental work that used a similar approach to ours^30^ found urchins were increasingly important when fish abundances were reduced. Thus, although the bulk of research on herbivory in coral reef ecosystems focuses on the role of fishes^5,10,14,14,15,22,24–27^, our research adds to a growing body of work elevating the ecological importance of urchin herbivory in maintaining reef resilience, particularly in ecosystems that have experienced substantial reductions in herbivorous fishes.

Urchin herbivory may become increasingly critical in a future with high sedimentation and intense overfishing. We found that urchin herbivory reduced sediment loads, likely through the mechanical action of consumption. Ample evidence demonstrates herbivorous fishes are deterred by sediment on turf^35,38–41^, suggesting that urchin herbivory, and the concomitant reduction in sediment load, likely facilitates fish herbivory on turf. With extreme precipitation events in the wet tropics predicted to become more intense and frequent^43^, it is likely that sedimentation will increase through increased transport and resuspension of sediments. As such, our results imply that the role of urchins in clearing sediment, cropping turf, and facilitating fish herbivory may become increasingly key to future reef resilience.

While our results highlight the potential for a key role for urchins in the resilience of future reefs, urchins are also significant bioeroders that can erode the reef framework, potentially preventing coral recovery. Overall, short turf increases the likelihood of a return to coral^16^, while taller turf can transition fully to macroalgae^15^. Thus, urchins may facilitate recovery if urchins do not limit early survivorship of coral recruits^52^. However, long term increases in bioerosion threaten this potential, as demonstrated on a degraded reef in the Galapagos, where high rates of bioerosion by urchins occurred in the absence of reef accretion^53^. While erosion rates have not been quantified in Moorea, gut analysis indicates bioerosion by urchins, on average, exceeds that of Labridae (subfamily Scarinae)^54^. Further, an individual urchin can erode substantial amounts of calcium carbonate, in some cases consuming more of the calcium carbonate substrate than the turf algae that grows on it^55,56^ . Compounded across individuals and time, erosion may shift a degraded reef from net accretion to net carbonate loss. Thus, while in the short-term urchins may enhance resilience of turf and allow recovery, if recruitment is low and corals do not recover, intensive urchin herbivory may threaten the reef framework, and reefs could be eliminated entirely.

## Methods

### Experimental site description and environmental context

**Figure 4:**
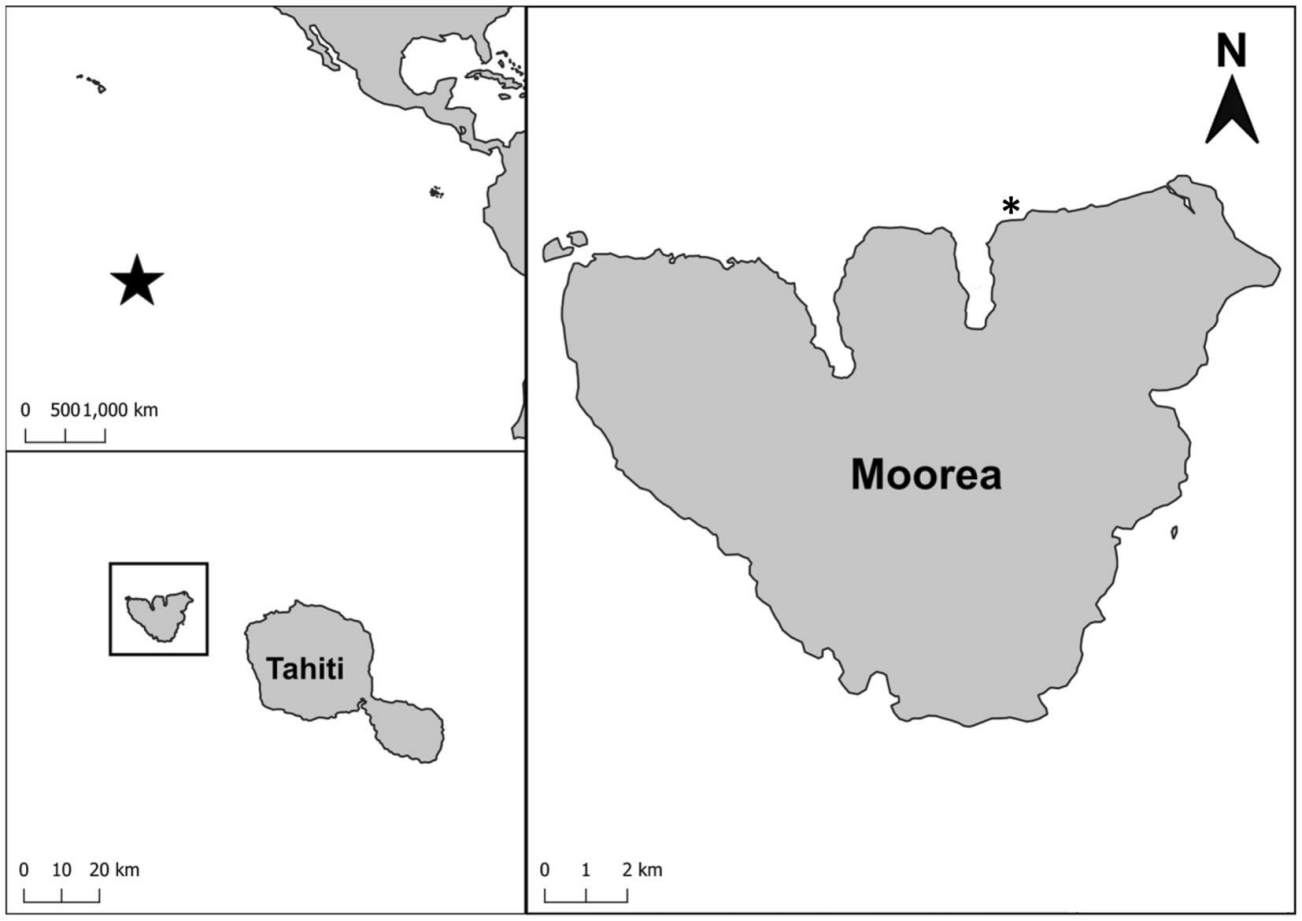
Maps showing the location of Moorea in the South Pacific and with respect to Tahiti. Close up map of Moorea with our site (and LTER fringing reef site 2) indicated with a * (17°28′57.78"S, 149°48′56.32"W).

We worked at Moorea, a tropical island in the South Pacific, part of French Polynesia’s Society Islands, and the site of the Moorea Coral Reef Long Term Ecological Research (MCR-LTER) program to leverage long term data to enrich and add context to our study. Specifically, we worked at Maharepa Reef (Fig. 4), or LTER 2, a shallow continuous fringing reef on the northern shore of the island consisting mostly of hard bottom with small sand patches. This reef is topographically complex with isolated dead *Porites* coral colonies that were killed during an *Acanthaster plancii* outbreak. We chose this fringing reef because, while forereef ecosystems of Moorea have largely recovered to coral dominance following crown of thorns outbreaks and a tropical storm between 2008–2012^57^, many fringing reefs have not recovered and remain dominated by turf algae^25,58^.

Our experiment occurred in the context of reduced herbivory and low sediment. This reef has comparably low biomass of herbivorous fishes^17^ and ambient sediment depths of <  ~ 0.1 mm, compared to 1.5 mm on more heavily-sedimented fringing reefs of Moorea^34^, both of which are lower than many values from the Great Barrier Reef^40^. Initial turf heights of 0.91 ± 0.05 mm (mean ± SE) confirmed a short turf community relative to others on Moorea^5,15,35^.

Our site is a long-term monitoring site (LTER 2) of the Moorea Coral Reef Long-Term Ecological Research (MCR LTER) Program. We used monitoring data collected by the LTER during the year, or as close to the year as available, of our experiment to characterize the benthos, the urchin community, and the herbivorous/detritivorous fish community at the site (we included the detritivore *Ctenochatetus striatus* as it consumes organic matter in turf sediment). Years for each data set are included in results.

### Conceptual framework and experimental design

Our experiment modeled 4 future overfishing scenarios crossed with 2 sedimentation regimes. Our first herbivory scenario is **status quo**, which models a future where the herbivore community remains unchanged. Our second scenario is **moderate overfishing and complete loss of urchins**, modeling a future where urchins are decimated by disease, as in the Caribbean in the 1980s, but where fishing pressure is not as severe as in the Caribbean at that time ^12^. Our third scenario models a reef with **severe overfishing but no loss of urchins**. This scenario is informed by the events in the Caribbean preceding the phase shift to macroalgal dominance, where the fish community was severely overharvested but urchin abundances retained^12^. Finally, we modeled **total loss of herbivory** of both herbivores, a ‘worst case scenario’ where herbivorous fishes are ecologically extinct and all urchins lost. Each of these herbivory scenarios were fully crossed with two sediment regimes, ambient and increased. The ambient sediment scenario models a future with the **status quo**, while the increased sediment scenario models a likely future where increased storms^43^ increase sediment resuspension and deposition on turf.

### Experimental set up

**Figure 5:**
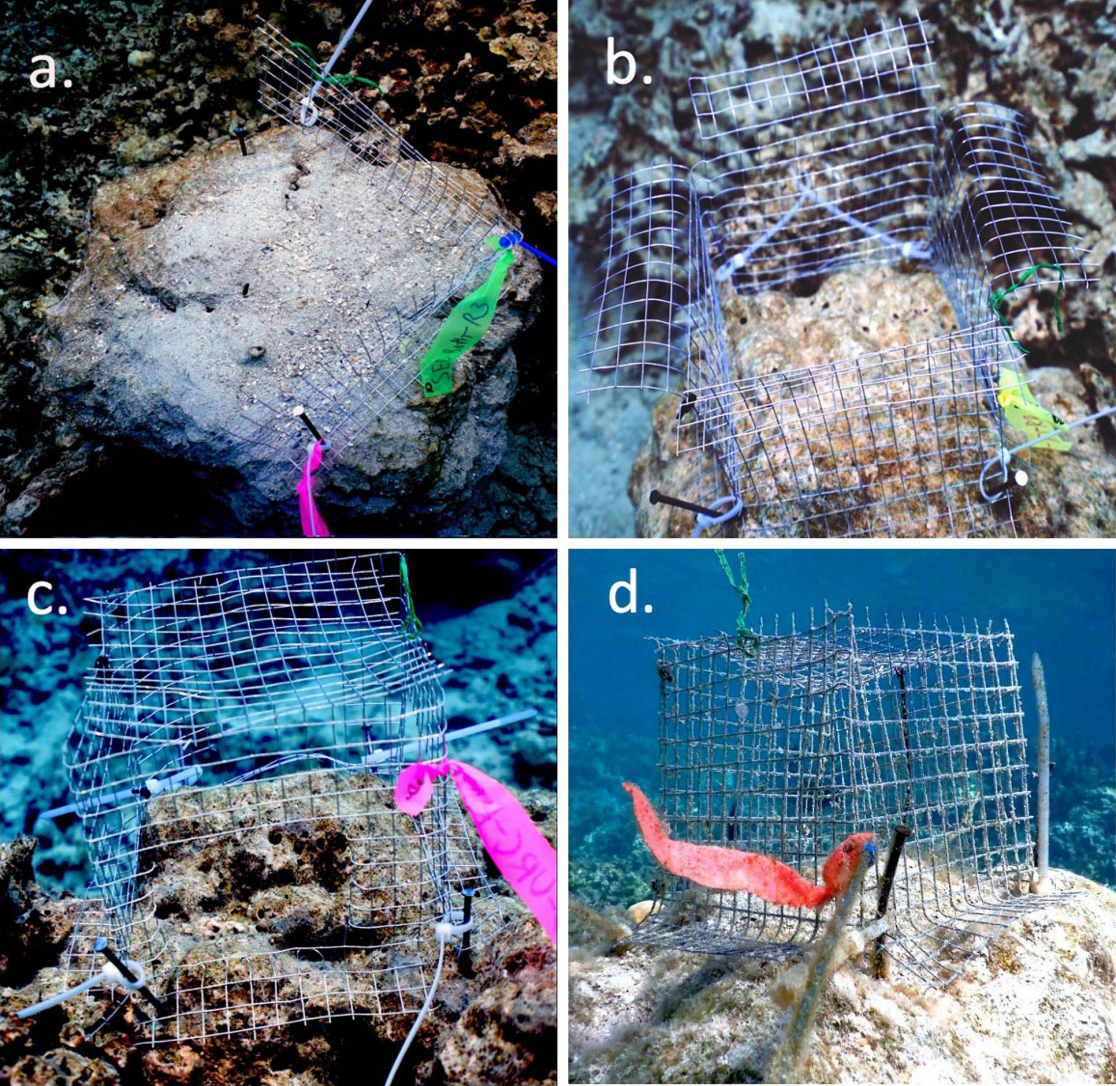
Cages to manipulate herbivore access. (**a**) Status quo, (**b**) moderate overfishing and loss of urchins, (**c**) severe overfishing but no loss of urchins, and (**d**) total loss of herbivory.

To model the four herbivory scenarios, we manipulated herbivore access with cages that resulted in different levels of access by urchins and herbivorous fishes, and then quantified access for herbivorous fishes for each cage type (see below). We enclosed 15 cm × 15 cm plots with full or partial cages constructed from hardware cloth with 1 cm openings, using different cage designs for different herbivory scenarios (Fig. 5). All plots were on top of dead coral heads between 1—4 m depth. Damselfish territories were excluded as they deter other herbivores^59^ and affect nutrient supplies^60^. To create our status quo scenario, we constructed a 2-sided barrier (15 cm × 15 cm; H × L) that allowed both fish and urchin entry while simultaneously providing a procedural control for changes in light and flow (*sensu*^38^, Fig. 5a). To create our moderate overfishing and loss of urchins scenario, we constructed a 4-sided cage with a 5 cm backward bending flange at the top to exclude urchins (*sensu*^29^). This cage was fixed flush to the benthos to eliminate access by urchins while simultaneously reducing access by herbivorous fishes (Fig. 5b). To create a scenario with severe overfishing but no loss of urchins, we constructed a 4-sided cage fixed flush to the benthos with a lid, but with panels cut out of the sides to allow urchin access while severely reducing fish access (Fig. 5c). Finally, to create a scenario of total loss of all herbivores > 1 cm, we constructed a 4-sided cage fixed flush to the benthos with a lid (Fig. 5d). All cages had 4 cm ‘skirts’ along their walls that were flush to the benthos to inhibit urchin entry^5,15,35^.

Our sediment scenarios modeled ambient versus increased sedimentation levels. The ambient sediment scenario was not manipulated. To simulate increased resuspension and deposition, we collected sediment adjacent to plots and supplemented half the plots with a depth of 3 mm, a level shown to negatively impact turf on another fringing reef of Moorea^35^. We ‘piped’ sediment onto the plot, and maintained this sediment depth, as needed, every 3 to 4 days to simulate a pressed sedimentation regime (*sensu*^35^).

Fully crossing four herbivory scenarios with two sedimentation levels created eight scenarios, which we replicated 10 times to total 80 units. After 15 days, we measured turf height and sediment depth to the nearest 0.5 mm with a wire mesh “comb” with teeth heights in 1 mm gradations (*sensu*^5^). We averaged turf height and sediment depth measured at 10 random points within each plot for a mean turf height and sediment depth for each replicate. We only collected sediment depth data for added sediment scenarios because initial data suggested ambient sediment load (no sediment added) was below our detection limit.

### Scenario efficacy

To validate efficacy of herbivore reduction scenarios, we observed each type of cage and quantified visitation rates of the dominant fish families (Acanthuridae and Labridae (subfamily Scarinae)) in situ for ambient sediment scenarios. We only quantified visitation for ambient sediment scenarios because sediment can deter herbivores^15,18,21,35,38^. On snorkel at a distance of at least four meters, we recorded the number of fish visits to each of the four cage types in 30-min focal intervals. We defined a visit as a fish entering the 15 cm × 15 cm × 15 cm cube of a plot. In total, we performed 25 30-min focals on our **status quo** scenario, 27 focals on our **moderate fish reductions and no urchins** scenario, 16 focals on our **severe fish reductions and full urchins** scenario, and 15 focals on our **no herbivory** scenario.

### Analyses

To test for differences in fish visitation rates across scenarios for Acanthuridae and Labridae (subfamily Scarinae), we used a Kruskal–Wallis analysis because data did not meet assumptions of parametric statistics even after transformation. We performed this analysis for families separately because we had no a priori expectation responses would be dependent. Significant Kruskal–Wallis results were followed with pairwise Wilcox tests with corrections for multiple comparisons. To determine how herbivory and sediment scenarios impacted turf height, we performed a 2-Way ANOVA on final turf height, which met assumptions of normality and homogeneity of variance after a log transformation. To determine how herbivory scenario impacted sediment depth, we performed a 1-Way ANOVA on final sediment depth (note, we did not measure final sediment depth for ambient sediment scenarios, as levels were so low as to be unmeasurable). These data met assumptions of normality and homogeneity of variance without transformation. We then conducted a Tukey HSD on all significant ANOVA results to determine which scenarios differed. Analyses were performed in base R version 4.1.2 (Fish_observations).

### Supplementary Information

Supplementary Information 1.

Supplementary Information 2.

Supplementary Information 3.

Supplementary Information 4.

Supplementary Information 5.

Supplementary Information 6.

## Data Availability

All data generated or analysed during this study are included in this published article [and its supplementary information files].
